# Spasmolytic Effect of Caulerpine Involves Blockade of Ca^2+^ Influx on Guinea Pig Ileum

**DOI:** 10.3390/md11051553

**Published:** 2013-05-13

**Authors:** Luiz Henrique Agra Cavalcante-Silva, Ana Carolina de Carvalho Correia, José Maria Barbosa-Filho, Bagnólia Araújo da Silva, Bárbara Viviana de Oliveira Santos, Daysianne Pereira de Lira, Jéssica Celestino Ferreira Sousa, George Emmanuel C. de Miranda, Fabiana de Andrade Cavalcante, Magna Suzana Alexandre-Moreira

**Affiliations:** 1 Laboratory of Pharmacology and Immunity, Institute of Biological Sciences and Health, Federal University of Alagoas, Maceió 57020-720, AL, Brazil; E-Mail: luiz0710@gmail.com; 2 Postgraduate Program in Natural Products and Synthetic Bioactive, Federal University of Paraíba, João Pessoa 58051-900, PB, Brazil; E-Mails: anacarolinacc@yahoo.com.br (A.C.C.C.); jbarbosa@ltf.ufpb.br (J.M.B.-F.); bagnolia@ltf.ufpb.br (B.A.S.); barbara@ltf.ufpb.br (B.V.O.S.); daysianneplira@yahoo.com.br (D.P.L.); jessicacelestino26@hotmail.com (J.C.F.S.); 3 Department of Pharmaceutical Sciences, Federal University of Paraíba, João Pessoa 58051-900, PB, Brazil; 4 Laboratory of Marine Algae, Department of Systematics and Ecology, Federal University of Paraíba, João Pessoa 58051-900, PB, Brazil; E-Mail: mirandag@dse.ufpb.br; 5 Department of Physiology and Pathology, Federal University of Paraíba, João Pessoa 58051-900, PB, Brazil

**Keywords:** caulerpine, guinea pig ileum, spasmolytic effect, Ca^2+^ channel

## Abstract

In this work, we investigated the spasmolytic effect of caulerpine, a bisindole alkaloid isolated from marine algae of the *Caulerpa* genus, on guinea pig ileum. Our findings indicated that caulerpine inhibited phasic contractions induced by carbachol (IC_50_ = 7.0 ± 1.9 × 10^−5^ M), histamine (IC_50_ = 1.3 ± 0.3 × 10^−4^ M) and serotonin (IC_50_ = 8.0 ± 1.4 × 10^−5^ M) in a non-selective manner. Furthermore, caulerpine concentration-dependently inhibited serotonin-induced cumulative contractions (pD′_2_ = 4.48 ± 0.08), shifting the curves to the right with *E*_max_ reduction and slope of 2.44 ± 0.21, suggesting a noncompetitive antagonism pseudo-irreversible. The alkaloid also relaxed the ileum pre-contracted by KCl (EC_50_ = 9.0 ± 0.9 × 10^−5^ M) and carbachol (EC_50_ = 4.6 ± 0.7 × 10^−5^ M) in a concentration-dependent manner. This effect was probably due to inhibition of Ca^2+^ influx through voltage-gated calcium channels (Ca_V_), since caulerpine slightly inhibited the CaCl_2_-induced contractions in depolarizing medium without Ca^2+^, shifting the curves to the right and with *E*_max_ reduction. According to these results, the spasmolytic effect of caulerpine on guinea pig ileum seems to involve inhibition of Ca^2+^ influx through Ca_V_. However, other mechanisms are not discarded.

## 1. Introduction

Natural products are a traditional source of drug molecules to treat infectious diseases, pain, and cancer, among others [1,2]. Marine organisms have played a relevant role among the natural products. They make many secondary metabolites with broad chemical diversity and complexity and with great pharmaceutical potential [3,4,5], such as conotoxins from cone snails [6] and compounds from algae [7] and tunicates [8]. Nowadays, there are four marine natural product-derived drugs approved for marketing: cytarabine and vidarabine, nucleosides with anticancer and antiviral properties, respectively; ziconotide, a peptide with potent analgesic activity; and trabectedin, a tetrahydroisoquinoline alkaloid approved for the treatment of soft tissue sarcoma and ovarian carcinoma [9].

Among the class of secondary metabolites in marine organisms, alkaloids are the second most abundant [10]. Indole alkaloids represent a quarter of all alkaloids [11], which are regarded as promising compounds in new drug discovery since they possess novel and complex frameworks [11,12]. Several activities are related to these compounds, including antiviral [13], cytotoxic [14], anti-inflammatory [15,16], antinociceptive [16], calmodulin antagonist [17], muscle relaxant [18], spasmolytic [19] and others.

**Figure 1:**
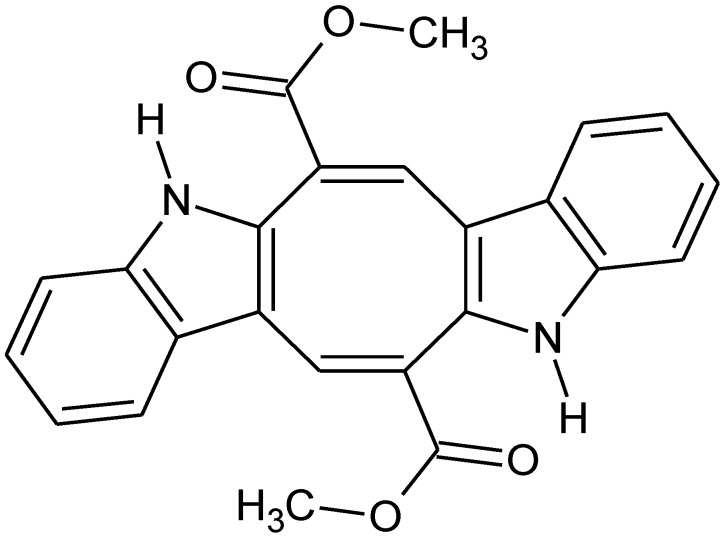
Structure of caulerpine.

5,12-dihydro-cycloocta[1,2-*b*;5,6-*b*′]diindole-6,13-dicarboxylic acid dimethyl ester, called caulerpine or caulerpin (Figure 1), is a bisindole alkaloid which is isolated mainly from green algae of the genus *Caulerpa* [20,21]. Additionally, its presence was also reported in other green (*i.e.*, *Codium decorticatum* and *Halimeda incrassate*) [22,23] and red (*i.e.*, *Chondria armata*) algae [24]. Since its first isolation in 1968 [20], caulerpine has shown some activities such as antitumor [25], antibacterial [26], inhibitor of human protein tyrosine phosphatase-1B (hPTP1B) [27], inhibitor of hypoxia-inducible factor (HIF) [28], antiviral [29], antinociceptive and anti-inflammatory [16].

Our research group reported some pharmacological activities of marine algae and their secondary metabolites [16,30,31,32]. Recently, we described the antinociceptive activity of caulerpine [16]. Since, Ca^2+^ is involved in the genesis of the nociceptive process [33] and Ca^2+^ signaling triggers smooth muscle contraction [34], besides the fact that other indole compounds have a spasmolytic effect [35,36,37], the aim of this work was to investigate the spasmolytic effect of caulerpine on guinea pig ileum.

## 2. Results and Discussion

In this work, the spasmolytic effect of caulerpine was evaluated in ileal smooth muscle of guinea pig. We demonstrated for the first time a non-selective spasmolytic effect of caulerpine, and that this effect is due in part to the inhibition of Ca^2+^ influx through voltage-gated calcium channels (Ca_V_).

**Figure 2:**
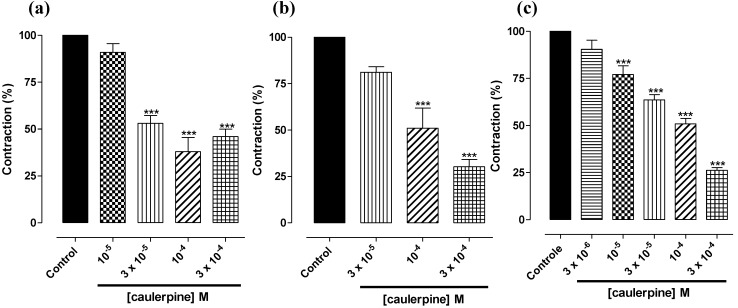
Effect of caulerpine on phasic contractions induced by 10^−6^ M CCh (**a**); 10^‑6^ M histamine (**b**); and 10^−5^ M serotonin (**c**); on guinea pig ileum (*n* = 5). Columns and vertical bars represent the means ± S.E.M., respectively. Significant differences are indicated by *** *p* < 0.001.

Initially, the caulerpine effect on phasic contractions induced by three different agonists was evaluated. It was observed that caulerpine antagonized phasic contractions induced by 10^−6^ M carbachol (CCh) (IC_50_ = 7.0 ± 1.9 × 10^−5^ M, *E*_max_ = 62.0 ± 7.6%), 10^−6^ M histamine (IC_50_ = 1.3 ± 0.3 × 10^−4^ M, *E*_max_ = 71.0 ± 4.9%) and 10^−5^ M serotonin (IC_50_ = 8.0 ± 1.4 × 10^−5^ M, *E*_max_ = 73.8 ± 1.4%) (Figure 2). A comparison of IC_50_ values showed that caulerpine had a non-selective effect. Silva *et al*. [35] has also reported a spasmolytic effect of another indole alkaloid, bisnordihydrotoxiferine, on rat uterus and guinea pig ileum.

On guinea pig ileum, the contractile effect of histamine and CCh are mediated by H_1_ [38] and M_3_ [39] receptors, respectively. Both receptors are coupled to the heterotrimeric G_q/11_ protein, which functions as a transducer to relay information to the inositol 1,4,5-trisphosphate (IP_3_)/diacylglycerol (DAG) signaling pathway, which in turn triggers an elevation in cytosolic calcium and consequent smooth muscle contraction [38,39,40]. On the other hand, serotonin-induced contraction is mediated by 5-HT_2A_ and 5-HT_3_ receptors [41,42]. The first is a G protein-coupled receptor (GPCR) that shares the same second messenger system with H_1_ and M_3_ receptors. 5-HT_3_ receptor is a ligand-gated ion channel and it is prone to rapid desensitization. The stimulation of 5-HT_3_ receptors on enteric cholinergic neurons induces acetylcholine release, resulting in smooth muscle contraction [41,42,43].

**Figure 3:**
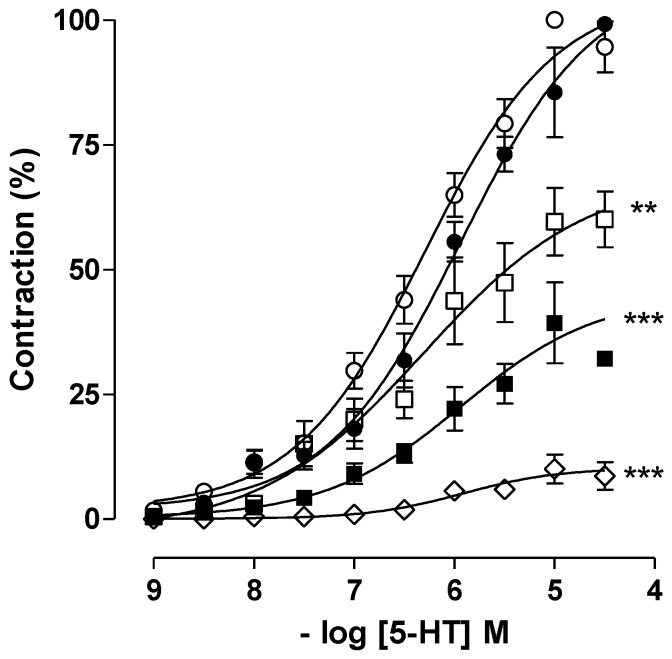
Cumulative concentration-response curves for 5-HT in the absence (○) and presence of caulerpine: 3 × 10^−5^ (●), 10^−4^ (□), 3 × 10^−4^ (■) and 10^−3^ M (◇) (*n* = 5). Symbols and vertical bars represent the means and S.E.M., respectively. One-way ANOVA followed by Bonferroni’s test, significant differences are indicated by ** *p* < 0.01 and *** *p* < 0.001.

Caulerpine antagonized phasic contractions induced by different agonists in an equipotent manner, which could suggest that this alkaloid does not act at the receptor level to inhibit contraction on guinea pig ileum. To confirm this hypothesis, serotonin-induced cumulative contractions were studied. As observed in Figure 3, caulerpine concentration-dependently inhibited serotonin-induced cumulative contractions and shifted the curves to the right with *E*_max_ reduction and slope of 2.13 ± 0.13, discarding thus a competitive type antagonism. The relaxation potency expressed as pD′_2 _was 4.48 ± 0.04 (Figure 2). Thus, by the antagonism pattern shown in Figure 3, the spasmolytic effect induced by caulerpine is characteristic of noncompetitive antagonism pseudo-irreversible. Furthermore, this result could explain the non-selective effect of caulerpine in relation to the agonists tested. Therefore, it is probable that caulerpine acts in another step of the cascade of events that leads to smooth muscle contraction. In addition, the spasmolytic effect on gastrointestinal smooth muscles through noncompetitive antagonism by the indole alkaloid trinervine was reported by Diniz *et al.* [36].

Guinea pig ileum shows biphasic contraction, comprising an initial phase in which the muscle exhibits a fast and transient contraction followed by a long-lasting second phase characterized by a maintained tonic contraction [44,45]. Both phasic and tonic contractions are dependent on extracellular calcium since both are inhibited in its absence [46]. The removal of extracellular Ca^2+^ prevents contraction induced by depolarizing agents, such as KCl, or by agonists, such as CCh and serotonin, in few seconds, indicating that intracellular Ca^2+^ does not contribute significantly to the tension level [47]. However, the influence of extracellular Ca^2+^ is relatively greater in tonic contractile response than in the phasic one [48]. Furthermore, the mechanisms involved in the maintenance of tonic contraction components are different from the phasic ones in guinea pig ileum [46]. Thus, the effect of caulerpine on the tonic contraction component induced by KCl (electromechanical coupling) and CCh (pharmacomechanical and electromechanical coupling) was investigated.

**Figure 4:**
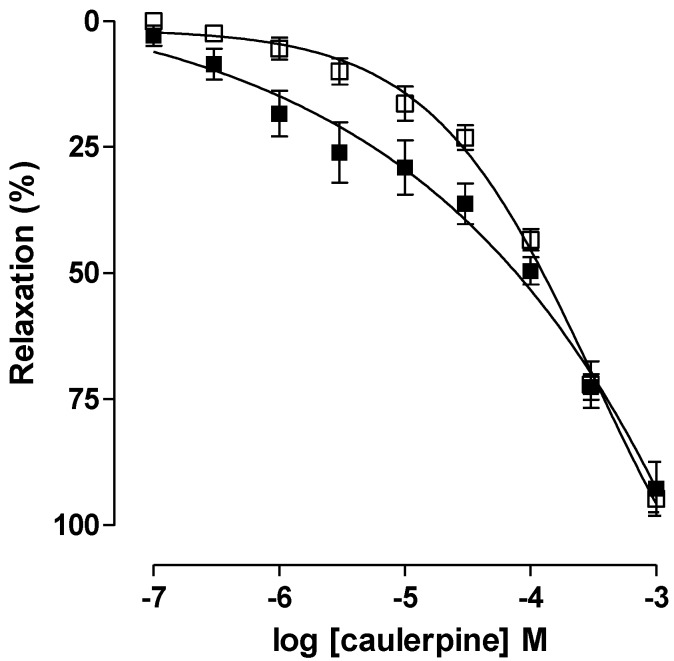
Effect of caulerpine on the tonic contractions induced by 40 mM KCl (□) and 10^‑5^ M CCh (■) on guinea pig ileum (*n* = 5). Symbols and vertical bars represent the means and S.E.M., respectively.

Caulerpine relaxed in a significant and concentration-dependent manner the ileum pre-contracted by 40 mM KCl (EC_50_ = 9.2 ± 0.9 × 10^−5^ M, *E*_max_ = 94.8 ± 2.6%) and 10^−5^ M CCh (EC_50_ = 5.2 ± 0.4 × 10^−5^ M, *E*_max_ = 92.7 ± 5.4) (Figure 4). An analysis of EC_50_ values indicated that caulerpine was slightly more potent in relax guinea pig ileum contracted by CCh than KCl, only 1.7-fold. The presence of an indole nucleus may be responsible for the relaxant effect, since a study performed with a series of derivatives analogous to *N*^b^-benzoyltryptamine (*N*-[2-(1*H*-Indol-3-yl)ethyl]benzamide), sharing the indole nucleus, showed non-selective relaxant activity on guinea pig ileum [37].

**Figure 5:**
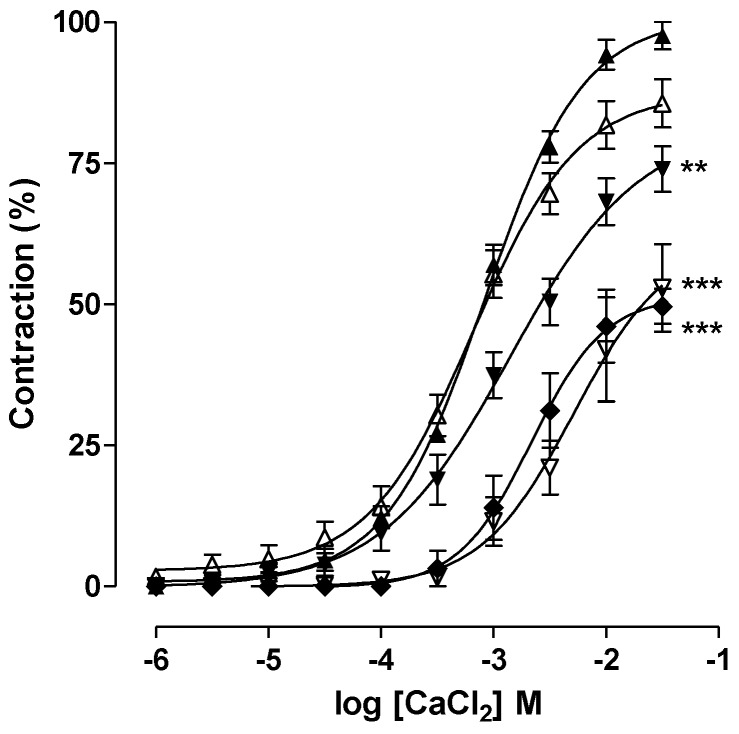
Cumulative concentration-response curves to CaCl_2_ in depolarizing medium nominally without Ca^2+^ in the absence (▲) and presence of caulerpine: 3 × 10^−5^ (△), 10^−4^ (▼), 3 × 10^−4^ (▽) and 10^−3^ M (♦). Symbols and vertical bars represent the means ± S.E.M., respectively. One-way ANOVA followed by Bonferroni’s test, significant differences are indicated by ** *p* < 0.01 and *** *p* < 0.001.

Independently of the contraction being evoked by either pharmacomechanical or electromechanical coupling, the maintenance of the tonic component involves the activation of Ca_V_ [49]. Therefore, we can postulate that caulerpine blocks these channels to produce non-selective spasmolytic effects. To evaluate this hypothesis, CaCl_2_-induced contraction in depolarizing medium nominally without Ca^2+^ was performed. This hypothesis was confirmed since CaCl_2_ cumulative concentration-response curves were shifted in a nonparallel way to the right and *E*_max_ was reduced from 100% (control) to 85.6 ± 4.2% (3 × 10^−5^ M), 73.9 ± 4.05% (10^−4^ M), 52.9 ± 7.7% (3 × 10^−4^ M) and 52.7 ± 4.5% (10^−3^ M) in the presence of caulerpine, which indicates a blockade of Ca^2+^ influx through Ca_V_ (Figure 5). Smooth muscle contractions are initiated when [Ca^2+^]_c_ attains a threshold level [40]. Thus, a decrease in [Ca^2+^]_c_ is a pivotal factor in smooth muscle relaxation.

Over the last six decades an enormous number of marine natural products have been studied. They are a valuable source of pharmacological tools, since they act on different and specific targets, and are thereby potentially useful leads in the development of new therapies [3,4]. In conclusion, we showed for the first time that caulerpine has a spasmolytic effect on guinea pig ileum through Ca^2+^ influx blockade. This makes such an alkaloid a potential compound for treating gastrointestinal disorders or a prototype to obtain new compounds acting on Ca^2+^ signaling.

## 3. Experimental Section

### 3.1. Isolation

*Caulerpa sertularioides* and *C. mexicana* algae were collected from the coastal region of Cabo Branco, João Pessoa, Paraíba State, Brazil in March 2009. The specimens were identified by Dr. George Emmanuel Cavalcanti de Miranda. Voucher specimens of *C. sertularioides* (JPB 13983) and *C. mexicana* (13985) have been deposited in the Lauro Pires Xavier Herbarium at the Federal University of Paraíba (Universidade Federal da Paraíba), Brazil. The alga was extracted with MeOH at room temperature and the extract was partitioned between hexane, dichloromethane, ethyl acetate and metanol. In the ethyl acetate phase there was precipitation of a orange red pigment. On the basis of its NMR spectral data and chemical properties, it was assigned the structure of 5,12-dihydro-cycloocta[1,2-*b*;5,6-*b*′]diindole-6,13-dicarboxylic acid dimethyl ester, named caulerpine or caulerpin.

### 3.2. Solutions and Drugs

Caulerpine was dissolved in Cremophor^®^ and diluted in distilled water. Carbamoylcholine chloride (carbachol), histamine dihydrochloride, serotonin hydrochloride were purchased from Sigma-Aldrich (St. Louis, MO, USA) and were dissolved and diluted in distilled water. The physiological solution was a freshly modified Krebs solution (pH 7.4) with the following composition (mM): NaCl (117.0), KCl (4.7), MgSO_4_ (1.3), NaH_2_PO_4_ (1.2), CaCl_2_ (2.5), glucose (11.0) and NaHCO_3_ (25.0). A high K^+^ isosmotic solution (pH 7.4) with the following composition was also used: NaCl (51.7), KCl (70.0), MgSO_4_ (1.3), NaH_2_PO_4_ (1.2), glucose (11.0) and NaHCO_3_ (25.0). These salts were obtained from Vetec (Rio de Janeiro, RJ, Brazil) and Fmaia (Cotia, SP, Brazil).

### 3.3. Animals

Adult guinea pigs (*Cavia porcellus*) of both sexes from the Professor Thomas George Bioterium of CBiotec/UFPB, weighing 368 ± 8 g, were used. The animals had free access to food and water, were kept in rooms at 21 ± 1 °C with a 12-h light–dark cycle and fasted for 18 h before the experiments. Measures to reduce pain, stress and any suffering were taken in accordance with the ethical guidelines for animal use. All experimental procedures were previously approved and performed in accordance with the Research Ethics Committee of the Federal University of Alagoas (UFAL) guidelines (protocol CEUA 039/2012).

### 3.4. Measurement of Contraction of Guinea Pig Ileum

Animals were euthanized by cervical dislocation and exsanguination. The distal ileum was immediately removed, cleaned of adhering fat and connective tissue, immersed in modified Krebs solution at room temperature and continuously gassed with carbogen (95% O_2_ and 5% CO_2_). Segments of the ileum oriented along the longitudinal axis (2–3 cm in length) were suspended in a 5 mL organ bath, which contained modified Krebs solution, maintained under resting load of 1.0 g at 37 °C. The tissues were allowed to stabilize for 30 min. Isotonic contractions were recorded using isotonic levers coupled to kymographs and smoked drums (DTF, Brazil) (Experiments 3.5.1 and 3.5.2). An isometric transducer (FORT-10) coupled to an amplifier (TMB4M), both from World Precision Instruments (EUA), connected to an analog/digital converter board (Bio Data, Brazil) installed in a computer with BioMed^©^ software version RV2 were used to record isometric contractions (Experiments 3.5.3 and 3.5.4).

### 3.5. Pharmacological Experiments

#### 3.5.1. Effect of the Caulerpine on CCh-, Histamine- and Serotonin-Induced Phasic Contractions

After a stabilization period, two phasic contractions were obtained for 10^−6^ M CCh, 10^−6^ M histamine or 10^−5^ M serotonin with intervals of 15 min between them. Caulerpine was then added, and after an incubation period of 15 min, a third concentration-response curve was obtained in the presence of various concentrations of caulerpine in different preparations. The procedure was repeated in the absence and in the presence of various concentrations of this alkaloid. The molar concentration of a substance that inhibits the response to an agonist by 50% (IC_50_) [50] was obtained by non-linear regression from the individual inhibition values for caulerpine.

#### 3.5.2. Effect of Caulerpine on Serotonin-Induced Cumulative Contractions

After a stabilization period, two similar cumulative concentration-response curves for serotonin were induced and caulerpine was incubated, in different preparations, in the absence of serotonin for 15 min in different concentrations as independent experiments. Afterwards, a new serotonin cumulative curve was obtained in the presence of caulerpine (3 × 10^−5^, 10^−4^, 3 × 10^−4^ and 10^−3^ M). The average amplitude of concentration-response curves for serotonin was considered to be 100% (control) and all contractions were assessed referring to it. Each preparation was exposed to only one caulerpine concentration. The antagonism exerted by caulerpine was evaluated based on the analysis of the Schild plot and their potencies with pD′_2_ values, which is defined as the negative logarithm to base 10 of molar concentration values of an antagonist that reduces the response to an agonist to 50% of its maximum effect (*E*_max_), assessed through concentration-response curves in both absence (control) and presence of caulerpine [51].

#### 3.5.3. Effect of the Caulerpine on KCl- or CCh-Induced Tonic Contractions

After a stabilization period, an isometric contraction was elicited by 40 mM KCl or 10^−5^ M CCh. Contractile agents remained in contact with the preparation until a contraction plateau was reached (approximately 10 min), and the tissue was then washed. After 30 min, the process was repeated and caulerpine was added cumulatively (10^−7^ up to 10^−3^ M) at the plateau phase. Relaxation was expressed as the reverse percentage of initial contraction elicited by the contractile agents. The molar concentration of a substance that produces 50% of its maximal effect (EC_50_) was obtained graphically from the concentration-response curves [50].

#### 3.5.4. Effect of Caulerpine on CaCl_2_-Induced Contractions in Depolarizing Medium Nominally without Ca^2+^

After the stabilization period, the modified Krebs solution was replaced by a depolarizing (with 70 mM KCl in equimolar exchange for NaCl) and nominally without Ca^2+^ solution for 45 min. Two similar CaCl_2_ cumulative concentration-response curves were obtained and caulerpine was incubated for 15 min in the absence of CaCl_2_. A third CaCl_2_ cumulative curve was obtained in the presence of caulerpine. The maximal contraction obtained with the first CaCl_2_ concentration-response curve was considered to be 100% (control), and all contractions were assessed in reference to it. Each preparation was exposed to a single caulerpine concentration.

### 3.6. Statistical Analysis

Data are expressed as means and S.E.M. EC_50_ and IC_50_ values were determined by nonlinear regression [50]. Differences between means were statistically compared using a *t*-test or one-way ANOVA followed by Bonferroni’s test when appropriate. The significance level considered in all tests was *p* < 0.05. Schild plots were analyzed by linear regression. Antagonism was considered to be noncompetitive when the slope of the Schild plot was significantly different from unity and depression of the maximum response was observed. All values were obtained using Graph-Pad Prism^®^ 5.01 software (GraphPad Software Inc., SanDiego, CA, USA).

## 4. Conclusions

In conclusion, we demonstrated that caulerpine has a non-selective spasmolytic effect on guinea pig ileum. At the functional level, this effect is due in part to the inhibition of Ca^2+^ influx through Ca_V_. However, other mechanisms cannot be excluded.
